# Human-Induced Pluripotent Stem Cells and Herbal Small-Molecule Drugs for Treatment of Alzheimer’s Disease

**DOI:** 10.3390/ijms21041327

**Published:** 2020-02-16

**Authors:** Wei Wuli, Sheng-Tzung Tsai, Tzyy-Wen Chiou, Horng-Jyh Harn

**Affiliations:** 1 Department of Bioinnovation Center, Buddhist Tzu Chi Medical foundation, Hualien 97002, Taiwan; dionysus2316@gmail.com; 2 Department of Life Science, National Dong Hwa University, Hualien 97401, Taiwan; 3 Department of Neurosurgery, Hualien Tzu Chi General Hospital, Hualien 97002, Taiwan; flydream.tsai@gmail.com; 4 Department of Pathology, Hualien Tzu Chi General Hospital, Hualien 97002, Taiwan

**Keywords:** induced pluripotent stem cells, Alzheimer’s disease, γ-Secretase

## Abstract

Alzheimer’s disease (AD) is characterized by extracellular amyloid plaques composed of the β-amyloid peptides and intracellular neurofibrillary tangles and associates with progressive declines in memory and cognition. Several genes play important roles and regulate enzymes that produce a pathological accumulation of β-amyloid in the brain, such as gamma secretase (γ-secretase). Induced pluripotent stem cells from patients with Alzheimer’s disease with different underlying genetic mechanisms may help model different phenotypes of Alzheimer’s disease and facilitate personalized drug screening platforms for the identification of small molecules. We also discuss recent developments by γ-secretase inhibitors and modulators in the treatment of AD. In addition, small-molecule drugs isolated from Chinese herbal medicines have been shown effective in treating Alzheimer’s disease. We propose a mechanism of small-molecule drugs in treating Alzheimer’s disease. Combining therapy with different small-molecule drugs may increase the chance of symptomatic treatment. A customized strategy tailored to individuals and in combination with therapy may be a more suitable treatment option for Alzheimer’s disease in the future.

## 1. Introduction

**Figure 1:**
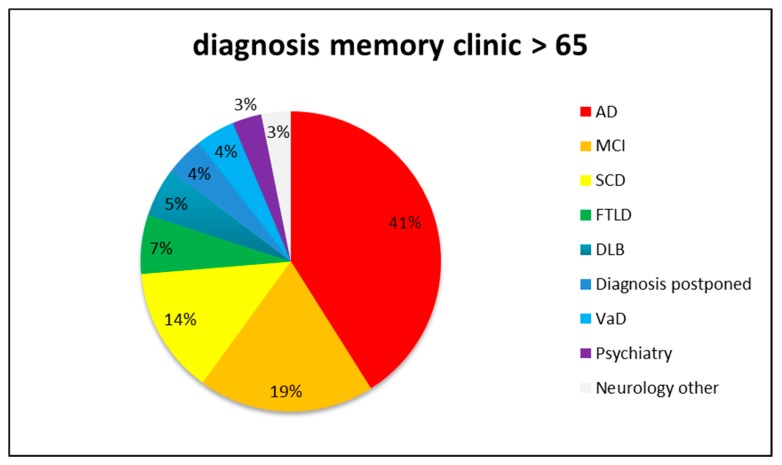
The proportion of dementia caused by various neurodegenerative diseases. AD = Alzheimer’s disease, MCI = mild cognitive impairment, SCD = subjective cognitive decline, FTLD = frontotemporal dementia, DLB = dementia with Lewy bodies, and VaD = vascular dementia.

Alzheimer’s disease is a prevalent neurodegenerative disease that worsens over time and causes brain lesions that can result in behavioral changes. It accounts for the largest number of dementia cases [1]. In the United States, the Alzheimer’s Association estimates approximately two new cases per year per 1000 people aged 65 to 74, 11 new cases per year per 1000 people aged 75 to 84, and 37 new cases per year per 1000 people aged 85 and older [2]. Alzheimer’s disease is characterizing by short-term memory loss accompanied by depression. Impairment of language ability leads patients to avoid difficult or complex words; the ability to recognize written characters is greatly reduced as well [3]. In the older age group (>65 years), Alzheimer’s disease is one of several dreaded neurodegenerative diseases that cause dementia. Others include frontotemporal dementia, dementia with Lewy bodies, vascular dementia, progressive supranuclear palsy, alcoholic dementia, Creutzfeldt–Jakob disease, and corticobasal degeneration (Figure 1) [4]. Alzheimer’s disease has two forms: early-onset and late-onset. Early-onset Alzheimer’s disease, also known as familial Alzheimer’s disease, occurs mainly before the age of 65 in association with hereditary genetic mutations. Late-onset Alzheimer’s disease, also known as sporadic Alzheimer’s disease, occurs after the age of 65, usually in association with environmental factors and age, and no inherited genetic mutation is known to be involved [5]. The early-onset form accounts for only about 5% of all patients with Alzheimer’s disease. However, gene expression is most directly related to early-onset Alzheimer’s disease [6]. Several gene mutations lead to increased accumulation of amyloid in the brain. The genes include *amyloid precursor protein (APP)*, *presenilin-1 (PSEN-1)*, and *presenilin-2 (PSEN-2)*. The *APP* gene sequence is located on human chromosome 21. APP protein was found in many tissues but is concentrated mainly in neuronal synapses. The functions of APP include the formation of regulatory synapses and the expulsion of iron atoms from cells [7]. APP protein exists in many different species and is highly conserved [8]. In humans, the *PSEN-1* gene sequence is located on chromosome 14, and the *PSEN-2* gene sequence is located on chromosome 1. *PSEN-1* and *PSEN-2* are highly homologous [9]. The function of PSENs is to form a complex with APP in cells and participate in the translocation and post-synthetic processing of APP [10].

Pathologically, AD is characterized by extracellular amyloid plaques composed of the β-amyloid peptides and intracellular neurofibrillary tangles that comprise the microtubule-associated protein tau [11]. Understanding the two pathologies that lead to neuronal death has been a focus of AD research. However, the generally used cell models of AD-PC12 and SH-SY5Y require the addition of Aβ1–42 peptides with induced cytotoxicity to mimic the symptoms of the disease [12,13,14,15,16,17]. However, the amyloid plaques are not the only symptoms of AD; hence, these in vitro research models are not comprehensive. The study of AD mechanisms in a human genetic context and natural display of AD symptoms in a cell model allow the understanding of AD pathology. Induced pluripotent stem cells (iPSCs) are a pathologically relevant in vitro model for the mechanistic studies and preclinical drug discovery of AD [18].

iPSCs are pluripotent stem cells formed by the lentiviral vector transfer of four transcription factors (i.e., *Oct4*, *Sox2*, *c-Myc*, and *Klf4*) into adult cells [19]. The benefits of iPSCs are that they differentiate into different types of neuronal cells from the stem cell stage and can be used to produce forebrain acetylcholine neurons, dopaminergic progenitor cells, and Purkinje cells [20,21,22,23]. Neuronal cells derived from iPSCs exhibit electrophysiological responses. Different types of iPSC-derived neurons have been used in the study of different diseases [24,25,26]. Basal forebrain cholinergic neurons degenerate in AD, Down syndrome, and dementia. Injectable acetylcholine agonists improve memory degradation in patients with AD [27]. Therefore, acetylcholine cells play a potentially regulatory role in memory and learning in the central nervous system [28]. The *platelet-derived growth factor promoter driven amyloid precursor protein* (*PDAPP*) transgenic mice exhibited amyloid deposition in the brain and memory dysfunction.

The cholinergic neurons derived from human iPSCs are transplanted to the hippocampus of *PDAPP* transgenic mice. Compared with vehicle-injected *PDAPP* mice, the transplantation of iPSC-derived differentiated neuronal precursor cells to *PDAPP* mice significantly improved memory dysfunction [29]. Purkinje cells are the only neurons in the cerebellar cortex that automatically produce two types of action potentials [30]. Damage to Purkinje cells is related to cognitive impairment and motor deficits in patients with AD [31]. The iPSC-induced increase in the number of Purkinje cell bodies is a potential novel strategy for AD treatment [22,32].

The AD cell model is useful for testing potential therapeutic drugs. Its use will increase the possibility of identifying an effective AD treatment through the collection of cells from patients with AD or Down syndrome and their reversal to iPSCs. The cells that have differentiated into neuronal cells and produce AD symptoms render them useful for exploring whether drugs are effective in the inhibition of these symptoms [33]. The use of iPSCs from patients with AD who have familial gene mutations will increase the possibility of developing effective therapeutic drugs, particularly those targeting the mechanisms of Aβ accumulation [34]. For example, a recent study using an iPSC platform exclusively derived from patients with AD was effective in the identification of small molecules and compounds with synergistic anti-Aβ effects [35]. Moreover, the collection of different early-onset AD gene mutations can produce different iPSCs based on AD symptoms. These approaches are helpful for understanding the mechanisms of AD and for developing drugs for this disease (Table 1).

## 2. Pluripotent Stem Cells Induced by AD Gene Mutations

### 2.1. APP Gene Mutations

Early-onset *APP* gene-mutated patient cells were used to produce a model of iPSCs overexpression, including a double mutation (KM670/671NL) of *APP*, which increases the total Aβ burden [36], followed by *APP* duplication to prepare iPSCs from patients with late-AD onset. The results revealed significantly higher levels of Aβ 1–40, phospho-tau (Thr 231), and active glycogen synthase kinase-3β (aGSK-3β) [37]. Interestingly, *APP* (A673T) mutations are protective against AD and cognitive decline [38].

### 2.2. Trisomy 21 Gene Mutation

Trisomy 21 is caused by the presence of an extra copy of the *APP* gene and is associated with increased Aβ peptide production. The accumulation of Aβ peptides is associated with a high risk of AD dementia in patients with Down syndrome [39]. iPSCs from individuals with trisomy 21 can induce Aβ aggregation, which causes increased expression of the tau protein and affects synapse formation and functionality [40].

### 2.3. PSEN1/2 Gene Mutation

Mutations of *PSEN1* and *PSEN2*, which are catalytic components of γ-secretase, are causative factors of autosomal dominant early-onset familial AD. Patients with familial AD have mutations in *PSEN1* (A246E) and *PSEN2* (N141I). Differentiated neurons derived from iPSCs of patients with familial AD exhibit increased toxic Aβ42 secretion [41]. Differentiated neurons derived from iPSCs with *PSEN1* (ΔE9) mutations can exhibit increased Aβ production, altered cytokine release, and deregulation of Ca^2+^ homeostasis [42].

### 2.4. Microtubule-Associated Protein Tau Gene Mutation

*Microtubule-associated protein tau* (*MAPT*) gene mutations are among the most common causes of familial frontotemporal dementia. *MAPT* (p.A152T) has been linked with frontotemporal dementia and Alzheimer’s disease. iPSCs from individuals carrying the tau-A152T variant of *MAPT* neurons showed accumulation, redistribution, and decreased solubility of tau. Upregulation of tau was coupled with enhanced stress-inducible markers and vulnerability of cells to proteotoxic, excitotoxic, and mitochondrial stressors [43]. iPSCs with *MAPT* mutations N279K, P301L, and E10 + 16 had neuritis outgrowth deficiencies [44]. The iPSCs were prepared separately, and their phenotypes manifested accumulations of tau protein, mitochondrial dysfunction, and defects of nerve growth and differentiation [45].

## 3. γ-secretase Inhibitors and γ-Secretase Modulators

**Figure 2:**
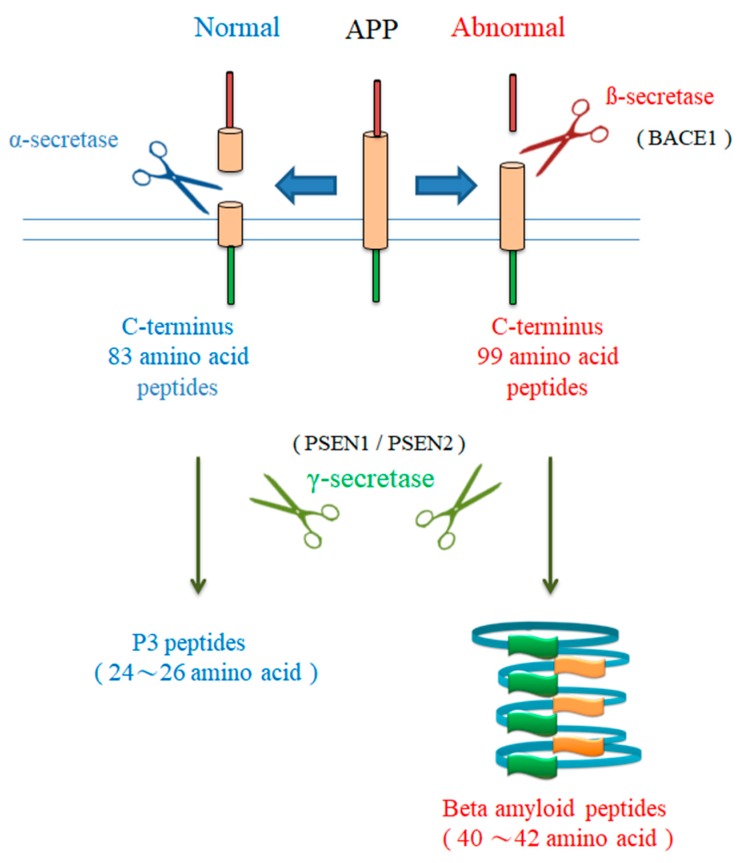
Beta-amyloid accumulation. In general, cleavage of beta amyloid precursor protein (APP) by alpha-secretase and γ-secretase cuts APP into P3 peptides (1–24~1–26 amino acids). However, once the gene generates mutations, such as *APP* and *GSIS1*, *PSEN1* gene expression increases, and the wrong cleavage is likely to occur and produce not easily metabolized peptide fragments (1–40~1–42 amino acids).

Mutations in the *PSEN-1* gene account for approximately 50% of the pathogenesis of early onset of AD and are the most common gene mutations in this disease [47]. Although gene mutations are responsible for early onset of AD, 95% of AD cases are late-onset diseases and are not necessarily caused by genetic mutations [48]. Late-onset AD can be caused by mutations in the *PSEN-1* gene; such mutations account for 25% of cases of late-onset AD [49]. The *PSEN-1* gene is an important regulator of γ-secretase in early- and late-onset AD (Figure 2). γ-secretase cleaves >90 different substrates. Studies of the γ-secretase-mediated cleavage of APP and Notch have focused on AD and cancer [50]. Complete inhibition of γ-secretase, as achieved by nonselective γ-secretase inhibitors (GSIs), abolishes all γ-secretase activity, leading to decreased Aβ production and Notch signaling. A recent publication used cryo-electron microscopy to elucidate the structures of the γ-secretase basis of the APP-C83 fragment and the structures of the γ-secretase basis of the Notch 100 fragment [51]. This will help in the development of substrate-specific γ-secretase inhibitors or modulators. Therefore, we discuss the development of GSIs and γ-secretase modulators (GSMs) in clinical settings.

### 3.1. γ-Secretase Inhibitors

**Figure 3:**
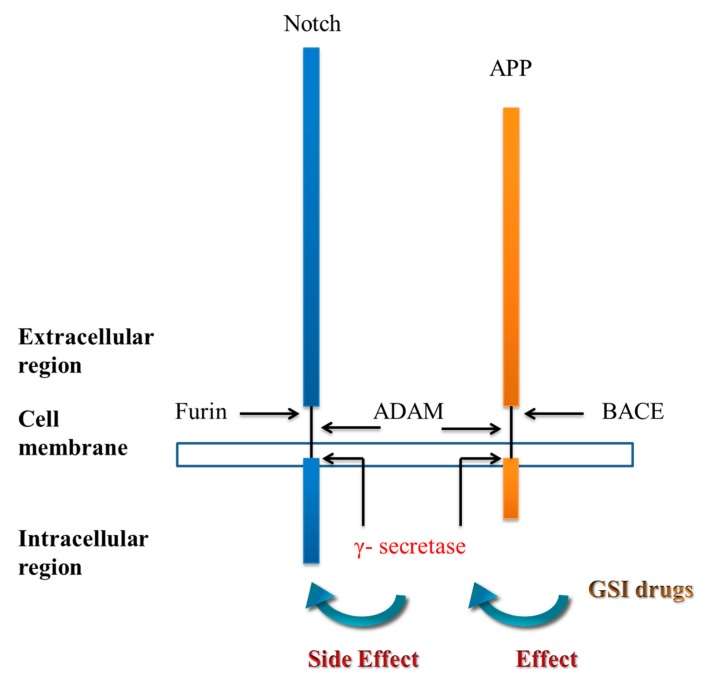
γ-secretase inhibitors. Notch and APP proteins are inactive when they are first synthesized and must have sections removed to become active. Furin and BACE first cleave these sections and activate the proteins. The second cleavage is by γ-secretase. GSIs can reduce γ-secretase but have a side effect of inhibiting Notch-signaling proteins. The function of this protein is related to nerve growth and differentiation. ADAM = A disintegrin and metalloproteinase, APP = amyloid precursor protein, BACE = beta-secretase, and GSI = γ-secretase inhibitors.

Most of the mutations in the *PSEN-1* gene are single nucleotide substitution mutations, which are missense mutations. *PSEN-1* mutations cause an increase in the production of amyloid-β protein (~90% 40-residue amyloid beta-protein and ~10% 42-residue amyloid beta-protein) [52,53,54]. PSEN-1 is one of the components of γ-secretase [55]. The drugs’ development strategies are through inhibition cleaved by γ-secretase and reducing the production of amyloid-β protein [56,57]. However, the mechanism to treat Alzheimer’s disease led to no improvement with cognitive function and accompanying adverse side effects led to failure [58]. Despite the initial failure of GSIs to treat Alzheimer’s disease successfully, these inhibitors are still considered to have potential. γ-secretase suppresses the expression of the intracellular domain of the Notch protein (NICD), which represents a failure of this strategy, because the Notch protein plays a key role in the function of adult neural stem cells [59], affecting the differentiation and proliferation of neurons [60]. Development of GSIs must account for the effect on the Notch signals (Figure 3), and GSIs can produce cell toxicity by increasing the levels of APP carboxy-terminal fragments (β-CTF) [61].

#### 3.1.1. Semagacestat

**Figure 4:**
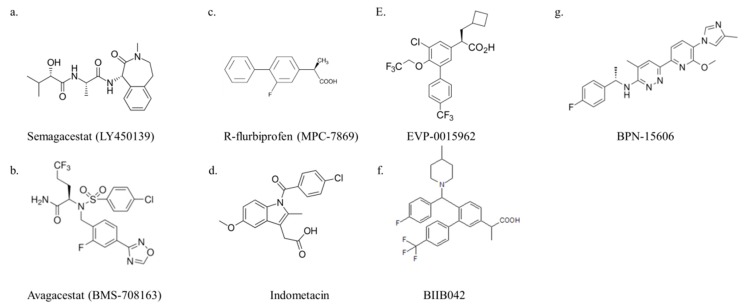
GSI and GSM structures. The GSIs are (**a**) semagacestat and (**b**) avagacestat. The first-generation GSMs are (**c**) R-flurbiprofen and (**d**) indomethacin. The second-generation GSMs are (**e**) EVP-0015962, (**f**) BPN-15606, and (**g**) BIIB042.

Semagacestat (LY-450139) (Figure 4a) was the first GSI to enter a phase III trial for AD. The compounds showed beta-amyloid-lowering effects in the in vitro assays [62]. Semagacestat is a γ-secretase inhibitor for Aβ42, Aβ40, and Aβ38 with IC50 of 10.9, 12.1, and 12.0 nM, respectively. It also inhibits Notch signaling with an IC50 of 14.1 nM in H4 human glioma cells [61]. The Tg2576 mouse model is an in vivo AD model. This model carries the *APP* KM670/671NL mutations. Oral administration of semagacestat (1 mg/kg) to 5.5-month-old *APP*-transgenic Tg2576 mice significantly improved the memory deficits [63]. Recently, reports have suggested that semagacestat is a pseudo-inhibitor of γ-secretase [64]. A phase III trial showed that semagacestat did not improve cognitive status and those patients receiving the higher dose exhibited significant deterioration of their functional ability. Semagacestat has more adverse effects, including cancers and skin infections (clinical trial number NCT00594568) [65].

#### 3.1.2. Avagacestat

Avagacestat (BMS-708163) (Figure 4b) was expected to enter a clinical phase II trial for AD. Avagacestat is a γ-secretase inhibitor of Aβ40 and Aβ42 with IC50 of 0.3 and 0.27 nM, respectively, and exhibits 193-fold selectivity against Notch in H4 human neuroglioma cells [66]. In vitro assays revealed that avagacestat reduced CSF Aβ levels without causing Notch-related toxicities [67]. The drug is administered orally to rats and dogs in the in vivo studies, and it significantly reduced Aβ40 levels for sustained periods in the brain, plasma, and cerebrospinal fluid [66]. A phase II clinical trial revealed that avagacestat was not effective (clinical trial number NCT00890890) [68].

γ-Secretase inhibitors (GSIs) block the γ-secretase-mediated cleavage of APP. However, these GSIs also block the γ-secretase-mediated cleavage of other substrates, including the critical signaling molecule Notch. For these reasons, drugs that do not inhibit the Notch signaling cleavage of γ-secretase modulators (GSMs) may be candidate treatments for AD [69]. Given the amyloid hypothesis for Alzheimer’s disease and ensuing cognitive decline, several treatments, including anti-beta-amyloid (Aβ) small organic molecules, antigens, and monoclonal antibodies, have been developed to interfere with Aβ production or increase clearance, including β-site beta-secretase (BACE) inhibitors, to block the first enzymatic step of the formation of Aβ or GSIs. However, these clinical trials targeting amyloid plaques of Alzheimer’s disease have failed in patients with mild-to-moderate Alzheimer’s disease [70]. GSIs have problems about leading to critical issues of Notch-related neuron toxicity [57,58]. Therefore, GSMs may be a better and safer strategy for drug development against AD. GSMs can act through an allosteric binding site to increase the concentrations of the shorter non-Aβ 1-42/Aβ 1-40 peptides.

### 3.2. γ-secretase modulators

**Figure 5:**
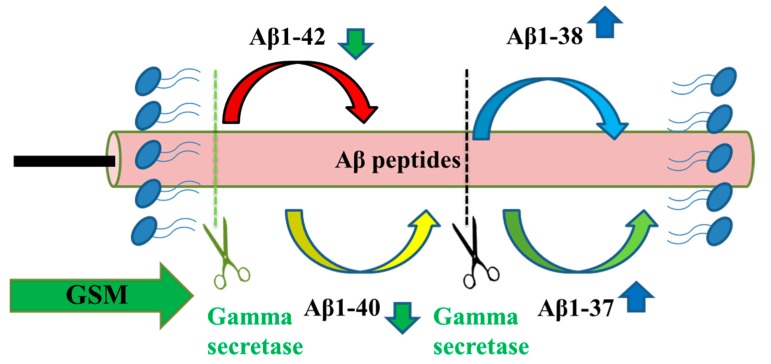
γ-secretase modulators. GSMs do not inhibit γ-secretase, but they can shift the profile of the secreted beta-amyloid peptides produced. GSMs can decrease the levels of Aβ1–42 peptides and increase the levels of Aβ1–38 peptides. Aβ = beta-amyloid and GSM = γ-secretase modulators.

GSMs differ from GSIs in that they do not alter total Aβ production, do not increase APP, and do not alter the generation of γ-secretase substrates but rather decrease Aβ1–42 peptides and increase Aβ1–38 peptides (Figure 5) [71]. Nonsteroidal anti-inflammatory drugs (NSAIDs), such as aspirin, is commonly used for pain and inflammation in adults. NSAIDs selectively reduce the formation of Aβ1-42 without inhibiting the proteolysis of Notch1. The first-generation GSMs include R-flurbiprofen and indomethacin (Figure 4c–g) [72].

#### 3.2.1. R-flurbiprofen (Tarenflurbil, MPC-7869)

R-flurbiprofen was expected to enter a clinical phase III AD trial (clinical trial number NCT00105547). R-flurbiprofen has functioned based on downregulation by neuronal tryptophan 2, 3-dioxygenase (TDO2). TDO2 expression was increased by neuronal cyclooxygenase-2 (COX-2) activities. The overexpression of TDO2 in the hippocampus led to behavioral deficits. R-flurbiprofen has prevented behavioral deficits in *APPSwe-PS1ΔE9* mice [73]. In clinical trials of patients with Alzheimer’s disease; R-flurbiprofen had low potency and brain penetration and was considered an unsuccessful treatment [74,75]. The shortcomings of the drug were not improvement by changes in the drug delivery route or the development of nanoparticle carriers [76].

#### 3.2.2. Indomethacin

Indomethacin was expected to enter a clinical phase III AD trial (clinical trial number NCT00432081). Indomethacin showed more inhibitory effects against COX-1 than against COX-2 [77]. Indomethacin attenuates neuronal pathological changes and cognitive impairments in APP/PS1 transgenic mice [78]. However, a clinical trial revealed that indomethacin did not improve the slow cognitive decline in patients with AD [79].

First-generation GSMs have low potency and inefficient blood–brain barrier penetrance, which affects the treatment of AD [80]. In second-generation GSMs, which include EVP-0015962, BIIB042 exhibited improved brain penetration ability [81]. GSM treatment of Alzheimer’s disease is considered feasible. BPN-15606 is a highly potent GSM that is a candidate for human clinical trials [82].

#### 3.2.3. EVP-0015962

EVP-0015962 (Figure 4e) decreased Aβ1–42 peptides and increased the shorter Aβ1–38 peptides, thus lowering Aβ42 in the H4 cell model. The total levels of Aβ and the amyloid precursor protein were not changed. In vivo, EVP-0015962 decreased memory deficits and reduced Aβ plaque formation in Tg2576 transgenic animals [83]. EVP-0015962 will enter a clinical phase II trial for the treatment of early AD onset (clinical trial number NCT01661673).

#### 3.2.4. BIIB042

BIIB042 (Figure 4f) is a potent γ-secretase modulator that lowered Aβ42, increased Aβ38, and did not affect Notch signaling in an in vitro model [84]. Tg2576 mice overexpress the human amyloid precursor protein and serve as a research model for AD. BIIB042 reduced Aβ42 levels and Aβ plaque burden in Tg2576 mice [85]. BIIB042 is a candidate drug for AD.

#### 3.2.5. BPN-15606

BPN-15606 (Figure 4g) inhibited the production of the Aβ1–42 and Aβ1–40 peptides, while facilitating the concomitant increase in Aβ1–38 and Aβ1–37 peptides production without inhibiting the γ-secretase-mediated proteolysis of Notch. BPN-15606 can lower Aβ42 levels significantly in the central nervous system of rats and mice [82]. Recent reports indicate that BPN-15606 is effective when administered prior to the severe pathological manifestations of AD [86].

## 4. Small-Molecular Combination GSM Treatments of Alzheimer’s Disease

**Figure 6:**
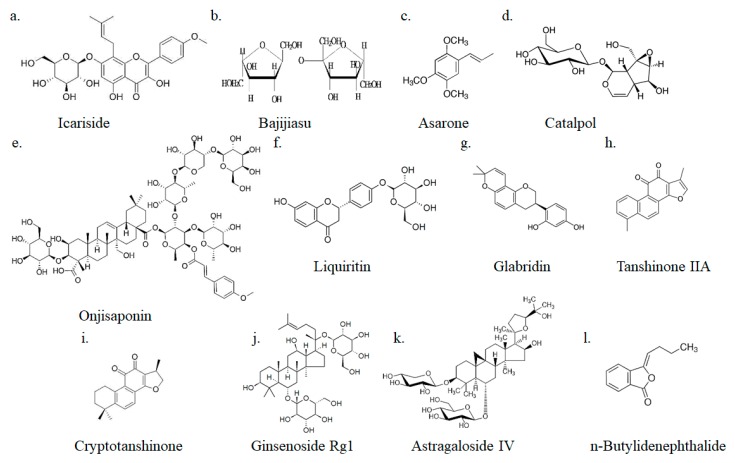
Small-molecule drug structures.

GSMs have the opportunity to become candidate drugs for the treatment of AD, but a combined treatment with drugs involving different mechanisms might provide additional efficacy for ameliorating cognitive impairment in Alzheimer’s disease. Many Chinese herbal medicines are considered to have the effect of treating dementia. We list small-molecule drugs extracted from commonly used Chinese herbal medicines and their mechanisms of treating Alzheimer’s disease (Table 2). The GSM structure is shown in Figure 6.

### 4.1. Icariside

*Icariside* is an extract from *Herba epimedii*, an ancient herbal medicine. Aggregates of Aβ are the agents responsible for disease progression in Alzheimer’s disease, and the accumulation of these aggregates is toxic to the brain. The major regulatory alpha-secretase in neurons, A disintegrin and metalloproteinase 10 (ADAM10), are responsible for cleaving APP. This cleavage generates a neuroprotective APP-derived short fragment (C83) and attenuates the production of Aβ peptides [87]. The mechanism of *icariside* in the treatment of Alzheimer’s disease involves increased ADAM10 expression and decreased expression of both APP and BACE1, resulting in reduced production of Aβ [88].

### 4.2. Onjisaponin

*Onjisaponin B* (*OB*) decreases Aβ by degradation of APP without direct inhibition of BACE1 and γ-secretase activities. In vivo, oral administration of *OB* mitigates Aβ pathology and behavioral defects in *APP/PS1* mice [89,90]. OB has effects of reduced IL-1β, IL-6, TNF-α, and malondialdehyde in the serum and hippocampus of lipopolysaccharide-induced cognitive-deficit rats. *OB* have beneficial effects by the antioxidant, anti-inflammatory, and anti-apoptosis in the lipopolysaccharide-induced PC12 cells [91].

### 4.3. Bajijiasu

*Bajijiasu* can inhibit neuronal inflammation, increase the levels of insulin degradation enzymes and neprilysin, and decrease BACE1 expression [92]. *Bajijiasu* have a function of neuron-protectiveness against Aβ-induced neurotoxicity in PC12 cells [93]. Animal studies also found the same function. In *APP/PS1* mice models, oral of *bajijiasu* improved learning and memory ability. The effects of *bajijiasu* have protected neurons against apoptosis, elevated the expression of neurotrophic factors, and alleviated endoplasmic reticulum stress [94].

### 4.4. Asarones

*Asarones*, the active constituents of *Acori Tatarinowii Rhizoma*, can improve the proliferation and self-renewal of neural progenitor cells. A mechanistic study showed that *asarones* activated extracellular signal-regulated kinase (ERK), part of a critical kinase cascade for neurogenesis [95]. *Asarone* have decreased Aβ deposition in the cortex and hippocampus of *APP/PS1* mice brains [96].

### 4.5. Catalpol

There is a significant positive correlation between brain-derived neurotropic factor (BDNF) content and memory. *Catalpol* can improve memory and reduce neurodegeneration by increasing BDNF expression [97]. In vitro, *catalpol* has been able to reduce the levels of soluble Aβ40 and Aβ42 in the cerebral cortex, and thus, inhibit the formation of senile plaques [98].

### 4.6. Liquiritin

*Liquiritin* have been extracted from *Glycyrrhiza uralensis*. Oxidative stress plays an important role in the pathogenesis of Alzheimer’s disease. Histopathological hallmarks of Alzheimer’s disease include formation of senile plaques composed of the Aβ peptides. Aβ can aggregate in formation with metal ions to catalyze production of reactive oxygen species [99]. Excessive oxidative stresses are generally regarded as a pathological condition. Malondialdehyde (MDA) and 8-hydroxy-2′deoxyguanosine (8-OHdG) are products of lipid peroxidation and are the most commonly detected biomarkers of oxidative stress [100]. *Liquiritin* reduces MDA and 8-OHdG in the hippocampus of rats with Alzheimer’s disease and, through related mechanisms, can significantly improve spatial learning and memory [101].

### 4.7. Glabridin

Deterioration of cholinergic innervation in the human cerebral cortex leads to significant cognitive and behavioral disorders in Alzheimer’s disease [102]. *Glabridin*, a small-molecule drug studied as a potential treatment for Alzheimer’s disease, has anticholinesterase activity. *Glabridin* reduced the brain cholinesterase activity and memory improvement in amnesia mice. The U.S. Food and Drug Administration approved cholinesterase inhibitors for the treatment of Alzheimer’s disease [103].

### 4.8. Tanshinone IIA and Cryptotanshinone

*Tanshinone IIA* (*TIIA*) and *cryptotanshinone* (*CT*) are extracted from *Salviae miltiorrhizae* as major lipophilic compounds. In vitro, *TIIA* and *CT* significantly reduced intracellular NO and Ca^2+^ levels in H9c2 cells [104]. The gliosis-related and neuro-inflammatory markers in the hippocampal tissues revealed a remarkable reduction in the expression of glial fibrillary acidic protein (GFAP), S100β protein, COX-2, inducible nitric oxide synthase (iNOS), and nuclear factor kappa-light-chain-enhancer of activated B cells (NF-kBp65) after CRY treatment. *TIIA* and *CT* display anti-inflammatory and neuroprotective effects in a nongenetic mouse model of AD [105].

### 4.9. Ginsenoside Rg1

*Ginsenoside Rg1* have been extracted from *Panax ginseng*. *Ginsenoside Rg1* repaired hippocampal long-term potentiation and memory in an Alzheimer’s disease mouse model. Through a mechanism that includes increased activity of the antioxidant enzymes glutathione peroxidase and superoxide dismutase and reduces astrocyte activation, it can increase the proliferation of hippocampal cells [106,107].

### 4.10. Astragaloside IV

Peroxisome proliferator-activated receptor (PPAR) agonists inhibit the expression of interleukin 6, tumor necrosis factor α, and cyclooxygenase 2 in microglial cells and monocytes stimulated by Aβ and reduce the proinflammatory and neurotoxic substances that these cells produce [108]. *Astragaloside IV* (*AS-IV*), derived from *Astragalus membranaceus*, is a natural PPARγ agonist that suppresses the activity of BACE1 and reduces the generation of Aβ. Activation of PPARγ in microglia by PPAR may be of value in the treatment of Alzheimer’s disease and associated inflammatory diseases [109].

### 4.11. n-Butylidenephthalide

*n-Butylidenephthalide* (*n-BP*) is derived from *Angelica sinensis*. It has been applied to the treatment of neurodegenerative diseases, including Parkinson’s disease [110], amyotrophic lateral sclerosis [111], and Alzheimer’s disease [40]. n-BP was shown to reduce Aβ, total tau protein and its hyperphosphorylated form, and cellular toxicity in iPSCs-derived neurons induced by Down syndrome [40].

## 5. Conclusions

**Figure 7:**
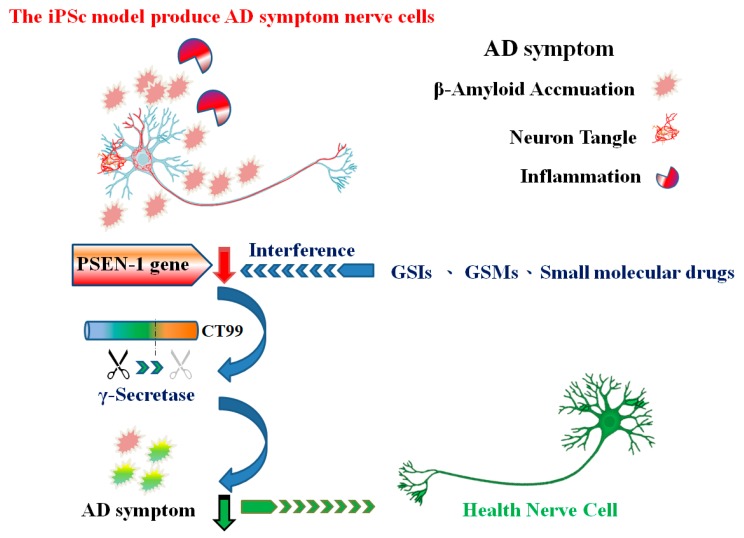
The iPSC screen drug strategy of Alzheimer’s disease. The AD-iPSC cell model can produces different AD symptoms, including β-amyloid accumulation, nerve entanglement, and inflammatory response. iPSCs have be customized according to personalization and, through various different treatment strategies, the treatment effect can be achieved more efficiently. AD = Alzheimer’s disease, GSI = γ-secretase inhibitors, GSM = γ -secretase modulators, and iPSC = induced pluripotent stem cell.

Recent studies report tau pathology and neurodegeneration are enhanced, but amyloid pathology is generally unaffected by concurrent tau pathology [113,114,115]. Through iPSCs, models get verified [116]. Using iPSCs that naturally produce Alzheimer’s disease symptoms to explore the mechanisms of small-molecule drugs and combining this approach with GSM or other therapies is a strategy for developing Alzheimer’s drugs. At the same time, using an iPSC platform for drug screening may be a good way to develop personalized medicine. Individual differences are perhaps the main cause of setbacks to the development of Alzheimer’s drugs. Establishing the patient’s iPSC to confirm the symptoms first and then accurately selecting the appropriate therapeutic drug may be a good way to provide effective treatment to patients while reducing the rate of failure of drug development. Through cryo-electron microscopy (cryo-EM) analysis, the structures for γ-secretase acting on the APP-C83 fragment and Notch-100 fragment will help develop substrate-specificity secretase inhibitors or modulators. Many small molecules extracted from Chinese herbal medicines, such as *Curcumin*, *Asarones*, and *Astragaloside*, have been used for the treatment of dementia [117,118,119]. However, these treatments have not achieved the expected results; thus, combinations of drugs with different mechanisms may be necessary. An increase in the number of choices may increase the chances of success in fighting Alzheimer’s disease (Figure 7).

## Figures and Tables

**Table 1:** Alzheimer’s diseases modeled with induced pluripotent stem (iPS) cells.

Gene Mutation of iPSCs	iPS Cells Produce Effect	References
*Presenilin-1* ΔE9 mutation	Impair γ-secretase activity but do not disrupt γ-secretase-independent functions of *PSEN1*	Minna Oksanen [42]
FAD patients with mutations in *PSEN1* (A246E) and *PSEN2* (N141I)	Increased toxic Aβ1-42 secretion	Takuya Yagi [41]
*Amyloid precursor protein* (*APP*) A673T mutation	Protective against Alzheimer’s disease and cognitive decline	Šárka Lehtonen [38]
*APP* double mutation (KM670/671NL)	Increase the total Aβ burden	Minna Oksanen [36]
Duplication of the *amyloid precursor protein* gene (*APP(Dp)*)	Increased Aβ 1–40, p-tau (Thr 231), and active glycogen synthase kinase-3β	Mason A. Israel [37]
Trisomy of chromosome 21 (Ts21)	Aβ aggregation and increased tau protein	Glenn A. Maclean [46]
A152T variant in *MAPT*	A152T-iPS cells-derived neurons showed accumulation, redistribution, and decreased solubility of tau.	M. Catarina Silva [43]
*MAPT* gene mutations (N279K, P301L, and E10+16)	Deficiencies in neurite outgrowth and upregulation of neurodegenerative pathways	Juan Antonio García-León [44]

**Table 2:** The role and mechanisms of small-molecule drugs treated in the Alzheimer’s disease model.

Extracts	Extracted from/Chinese Name	Drug Doses & Experimental Model	Possible Molecular Mechanism Effect	Reference
*Icariin II*	*Herba Epimedii*/淫羊藿	*APP/PS1* transgenic mice were treated orally g *icariside II* 30 m/kg.	Effectively ameliorated cognitive function deficits, but also inhibited neuronal degeneration and reduced the formation of plaque burden.	[88]
*Onjisaponin B*	*Radix polygalae*/遠志	*APP/PS1* transgenic mice were treated orally 200 μL of *onjisaponin B* 1 mg/mL.	The *Onjisaponin B* suppresses Aβ production without direct inhibition of β-secretase and γ-secretase activities.	[89]
*Bajijiasu*	*Morinda officinalis*/巴戟天	*APP/PS1* transgenic mice were treated orally 20 mg and 80 mg *bajijiasu*/kg.	Reductions of Aβ deposition and senile plaques and have higher levels of neurotrophic factors and inhibitory function on neuroinflammation in the brains of *APP/PS1* mice.	[92]
*Asarones*	*Rhizoma Acori tatarinowii*/石菖蒲	Adult hippocampal neural progenitor cells (NPC) cultures treated with 1 μM *asarones*.	The *Asarones* enhanced NPC proliferation and neurogenesis in the hippocampi of transgenic AD model mice.	[95]
*Catalpol*	*Rehmannia glutinosa*/地黃	*APP/PS1* transgenic mice were treated orally *catalpol* 50 mg / kg.	Improves memory and protects the forebrain neurons through increasing BDNF expression.	[97]
*Liquiritin*	*Glycyrrhizae radix*/甘草	Soluble amyloid-β1-42 oligomers injected into the hippocampus induced cognitive-deficit rats. Treated by *liquiritin* was orally 50~100 mg/kg.	The drugs improve Aβ1-42-induced spatial learning and memory impairment through inhibiting oxidative stress and neural apoptosis.	[101]
*Glabridin*	*Glycyrrhiza glabra*/洋甘草	Oral *glabridin* 25 and 50 mg/kg treated for diabetic rats [112].	It reversed learning and memory deficits of diabetic rats.	[103]
*Tanshinone IIA*	*Salviae miltiorrhizae*/丹参	Oral 10mg/kg treated for nongenetic mouse model of β-amyloid-induced AD.	That *TIIA* and *CT* display anti-inflammatory and neuron-protective effects in a nongenetic mouse model of AD.	[105].
*Cryptotanshinone*				
*Ginsenoside Rg1*	*Radix ginseng*/人參	Oral 20 mg/kg D-galactose-induced aging rat model.	It prevents cognitive impairment and hippocampus senescence of D-galactose-induced aging in a rat model.	[107]
*Astragaloside IV*	*Astragalus membranaceus*/黃耆	*APP/PS1* mice that were treated orally *AS-IV* 10 mg/kg.	*AS-IV* treatment increased PPARγ and reduced Aβ plaque formation in the brain.	[109]
*n-Butylidenephthalide*	*Angelica sinensis*/當歸	Down syndrome - iPSCs were treated *n-BP* 10 μM for 3 days.	*n-BP* benefitted AD treatment by scavenging Aβ aggregates and neurofibrillary tangles.	[40]

## Abbreviations

AD	Alzheimer’s disease
ADAM	A disintegrin and metalloproteinase
APP	Amyloid precursor protein
AS-IV	Astragaloside IV
BACE	β-site beta-secretase
BDNF	Brain-derived neurotropic factor
cryo-EM	Cryo-electron microscopy
COX-2	Cyclooxygenase-2
CT	Cryptotanshinone
DLB	Dementia with Lewy bodies
ERK	Extracellular signal-regulated kinase
FTLD	Frontotemporal dementia
GSI	γ-secretase inhibitors
GSMs	γ-secretase modulators
GFAP	Glial fibrillary acidic protein
iNOS	Inducible nitric oxide synthase
MAPT	Microtubule-associated protein tau
MCI	Mild cognitive impairment
MDA	Malondialdehyde
NICD	Notch intracellular domains
NSAIDs	Nonsteroidal anti-inflammatory drugs
NF-kBp65	Nuclear factor kappa-light-chain-enhancer of activated B cells
n-BP	n-Butylidenephthalide
OB	Onjisaponin B
PSEN-1	Presenilin-1
PSEN-2	Presenilin-2
PDAPP	PDGF promoter driven amyloid precursor protein
PPAR	Peroxisome proliferator-activated receptor
SCD	Subjective cognitive decline
TIIA	Tanshinone IIA
VaD	Vascular dementia
8-OHdG	8-hydroxy-2′deoxyguanosine
